# Bioremediation of aflatoxin B1-contaminated maize by king oyster mushroom (*Pleurotus eryngii*)

**DOI:** 10.1371/journal.pone.0182574

**Published:** 2017-08-03

**Authors:** Maria Teresa Branà, Maria Teresa Cimmarusti, Miriam Haidukowski, Antonio Francesco Logrieco, Claudio Altomare

**Affiliations:** 1 Institute of Sciences of Food Production, National Research Council of Italy, Bari, Italy; 2 Department of Economics, University of Foggia, Foggia, Italy; Leibniz-Institut fur Pflanzengenetik und Kulturpflanzenforschung Gatersleben, GERMANY

## Abstract

Aflatoxin B1 (AFB_1_) is the most harmful mycotoxin that occurs as natural contaminant of agricultural commodities, particularly maize. Practical solutions for detoxification of contaminated staples and reduction of agricultural wastes are scarce. We investigated the capability of the white-rot and edible fungus *Plerotus eryngii* (king oyster mushroom) to degrade AFB_1_ both *in vitro* and in a laboratory-scale mushroom cultivation, using a substrate similar to that routinely used in mushroom farms. In malt extract broth, degradation of AFB_1_ (500 ng/mL) by nine isolates of *P*. *eryngii* ranged from 81 to 99% after 10 days growth, and reached 100% for all isolates after 30 days. The growth of *P*. *eryngii* on solid medium (malt extract-agar, MEA) was significantly reduced at concentrations of AFB_1_ 500 ng/mL or higher. However, the addition of 5% wheat straw to the culture medium increased the tolerance of *P*. *eryngii* to AFB_1_ and no inhibition was observed at a AFB_1_ content of 500 ng/mL; degradation of AFB_1_ in MEA supplemented with 5% wheat straw and 2.5% (w/v) maize flour was 71–94% after 30 days of growth. Further, AFB_1_ degradation by *P*. *eryngii* strain ITEM 13681 was tested in a laboratory-scale mushroom cultivation. The mushroom growth medium contained 25% (w/w) of maize spiked with AFB_1_ to the final content of 128 μg/kg. *Pleurotus eryngii* degraded up to 86% of the AFB_1_ in 28 days, with no significant reduction of either biological efficiency or mushroom yield. Neither the biomass produced on the mushroom substrate nor the mature basidiocarps contained detectable levels of AFB_1_ or its metabolite aflatoxicol, thus ruling out the translocation of these toxins through the fungal thallus. These findings make a contribution towards the development of a novel technology for remediation of AFB_1_- contaminated corn through the exploitation of the degradative capability of *P*. *eryngii* and its bioconversion into high nutritional value material intended for feed production.

## Introduction

Aflatoxin B_1_ (AFB_1_) is a mycotoxin, produced mainly by isolates of the species *Aspergillus flavus* and *A*. *parasiticus*, which has potent hepatotoxic, carcinogenic and mutagenic effects on humans and animals [1]. Beside AFB_1_, other aflatoxins that are structurally correlated to AFB_1_, occur as natural contaminants of foods and feeds or are generated from the metabolic transformation of AFB_1_, but have considerably lower incidence and toxicity than AFB_1_ [2]. AFB_1_ has been listed as a group I agent (carcinogenic to humans) by the International Agency for Research on Cancer and epidemiological studies have correlated the incidence of hepatocellular carcinoma in humans to consumption of AFB_1_-contaminated food in some world regions [3]. Human exposure to aflatoxin can result directly from ingestion of contaminated food or indirectly from consumption of products from animals that have been fed with contaminated feed. As a result of ingestion of such feeds, aflatoxins are transformed into metabolites that contaminate meat, eggs and dairy products, such as milk and cheese [4]. Aflatoxin occurrence is a major problem in a number of crops, including cereals, groundnuts, legumes and cotton seeds, which can be contaminated at any stage of production, processing, transportation, and storage [5]. Amongst the cereal grains, aflatoxin contamination concerns primarily maize and maize by-products [6].

Several approaches have been attempted for the removal of aflatoxins from contaminated commodities, including degradation of the toxin [7]. Degradation of aflatoxin requires the alteration of one or both of the molecule sites that are important for its toxic properties, namely the double bond of the difuran ring and the lactone ring of the coumarin moiety [8].Chemical and physical methods have been found to be effective in detoxification of AFB_1_ from various materials [9,10], but their use in the practice is limited, due to safety issues, possible loss of nutritional value of the treated commodities and cost implications [11]. Microbial degradation of aflatoxins has been attempted with some success, although in most cases only in axenic cultures, [12–17]. The microbial degradation of aflatoxin is achieved by the activity of enzymes able to break down the recalcitrant polyheterocyclic molecule of aflatoxin. Among fungi, the so called “white-rot” fungi are known to possess very efficient enzymatic systems for degradation of polycyclic aromatic hydrocarbons [18]. Indeed, the enzymes produced by the white-rot fungi have the crucial role of breaking down the complex molecules of lignin and other plant raw materials into low molecular weight compounds that can be assimilated by the organism. This process involves multiple ligninolytic enzyme systems consisting in extracellular oxido-reductases [19]. Encouraging results in aflatoxin degradation have been obtained with specific enzymes purified from *Pleurotus* spp., a genus that includes several edible and cultivable mushroom species. Motomura and colleagues [20] reported degradation of AFB_1_ by culture supernatants of *P*. *ostreatus* and isolated a novel enzyme with aflatoxin-degradation activity. More recently, Yehia [21] has shown that a Mn-peroxidase (MnP) purified from *P*. *ostreatus* was able to detoxify up to 90% of AFB_1_, depending on enzyme concentration and exposure time. Other enzymes produced by *Pleurotus* spp. that have received attention because of their aflatoxin-degradative capability are laccases, a group of enzymes of low specificity that catalyse the oxidation of phenolic substrates *via* the reduction of molecular oxygen to water. Alberts and colleagues [22] reported a significant correlation between laccase activity and AFB_1_-degradative capability of *P*. *ostreatus* isolates. Loi and colleagues [23] purified a laccase isoform (Lac2) from *P*. *pulmonarius* and found that AFB_1_ degradation by Lac2 *via* direct oxidation was 23%, which raised up to 90% in the presence of natural phenols acting as redox mediators.

*Pleurotus eryngii* is a cultivable edible white-rot fungus, commonly known as king oyster mushroom (KOM). The KOM is cultivated worldwide and is highly appreciated for its firm texture, taste, flavor and nutritional value [24]. The goals of the work herein presented were: a) investigate the capability of *P*. *eryngii* strains that are exploited for commercial production of edible mushrooms to degrade AFB_1;_ b) carry out a study on bioconversion of aflatoxin-contaminated maize into valuable feeds by *P*. *eryngii* in a laboratory-scale cultivation that mimicked the mushroom farm cultivation process; and c) assess the aflatoxin content in the residual mushrooms growth medium and investigate translocation of AFB_1_ or its toxic metabolite aflatoxicol (AFOL) through the thallus to the basidiocarps (fruit bodies) of *P*. *eryngii*. The results obtained make a first contribution towards the development of a novel technology for remediation of AFB_1_-contaminated corn and its bioconversion into safe materials intended for feeds.

## Materials and methods

### Fungal strains

The nine isolates of *P*. *eryngii* used in the study are strains commercially exploited for production of mushrooms. The isolates were obtained from the culture collection of the Institute of Sciences of Food Production (ITEM Collection, http://www.ispa.cnr.it/Collection/), Bari, Italy and were characterized as belonging to the variety *eryngii* (strains ITEM 13662, ITEM 13676, ITEM 13681, ITEM 13682, ITEM 13696, ITEM 13697, ITEM 13688, ITEM 13730, ITEM 17015) or variety *ferulae* (ITEM 13688) by sequencing of partial regions of the genes *ef1-*α and *rpb2* [25, Susca A. personal communication, 2016]. The cultures were maintained in purity on malt extract agar (MEA, Oxoid, Basingstoke, UK) slants, which were used as sources of inocula for subsequent cultures.

### Determination of aflatoxin B_1_ and aflatoxicol

The standard solution of AFB_1_ was prepared by dissolving the solid commercial toxin (Sigma-Aldrich, Milan, Italy) in toluene-acetonitrile (9:1, v/v) into amber silanized vials to obtain a 1 mg/mL solution. The exact concentration of aflatoxin solution was determined spectrometrically according to AOAC Official Method 971.22 [26]. Aliquots of the stock solution were transferred to 4 mL amber silanized glass vials and evaporated to dryness under a stream of nitrogen at 50°C. The residue was dissolved with water-methanol (60:40, v/v) to obtain calibrant standard solutions with 0.2, 0.4, 1.2, 2.0, 4.0, 5.0, 10.0 ng/mL of AFB_1_. The standard solution of AFOL, Sigma-Aldrich, Milan, Italy) in acetonitrile was transferred to amber silanized glass vials and dried under a stream of nitrogen at 50°C, then was reconstituted with water-methanol (50:50, v/v), to obtain calibrant standard solution with 2.0, 5.0, 10.0, 25.0, 50.0, 100 ng/mL of AFOL. Standard solutions were stored at -20°C and warmed to room temperature before use.

AFB_1_ was determined by using UPLC apparatus with Acquity UPLC system (Waters, Milford, MA, USA). Data acquisition and instrument control were performed by Empower 2 software (Waters). The column used was a 100 mm × 2.1 mm i.d., 1.7 μm, Acquity UPLC^®^ BEH RP-18, with an Acquity UPLC column in-line filter (0.2 μm), detected by fluorometric detector without postcolumn derivatization. The fluorometric detector was set at wavelengths of 365 nm (excitation) and 435 nm (emission). The mobile phase was a mixture of water-acetonitrile-methanol (64:18:18, v/v/v) at a flow rate of 0.4 mL/min. The temperature of the column was maintained at 40°C. AFB_1_ was quantified by measuring the peak areas at the retention time of aflatoxin standard and comparing these areas with the calibration curve of AFB_1_ in the range 0.2 to 10.0 ng/mL. With this mobile phase, the retention time of AFB_1_ was about 3.7 min. The limit of quantification (LOQ) of the method was 0.2 ng/mL for AFB_1_, based on a signal to noise ratio of 10:1.

Analyses of AFOL were performed with a HPLC Agilent 1260 Series (Agilent Technology, SantaClara, CA, USA) with post column photochemical derivatization (UVE^™^, LC Tech GmbH, Dorfen, Germany). The analytical column was a Luna PFP (150 × 4.6 mm, 3 μm) (Phenomenex, Torrance, CA, USA) preceded by a Security Guard^™^ (PFP, 4×3.0 mm, Phenomenex). One hundred microliters samples were injected into the HPLC apparatus with a full loop injection system. The fluorometric detector was set at wavelengths of 333 nm (excitation) and 418 nm (emission). The mobile phase consisted of a mixture of H_2_O-ACN (70:30, v/v) and the flow rate was 0.8 mL/min. The temperature of the column was maintained at 40°C. AFOL was quantified by measuring peak areas at the retention time of standard solutions and comparing these areas with the relevant calibration curve at 2.0–100 ng/mL. In this analytical conditions, the retention time was about 16 min. The limit of quantification (LOQ) values were 20 μg/kg, calculated in according to s/n = 10.

### Stability of AFB_1_ in malt extract broth

A preparatory study was carried out to assess the decay of AFB_1_ in the incubation conditions utilized for the subsequent experiments. AFB_1_ was dissolved in malt extract broth (MEB) (Oxoid) to the final concentration of 1000 ng/mL and the solution was placed at 30 ± 1°C for 30 days. Triplicate 1 mL samples were collected from the batch solution of AFB_1_ after 0, 1, 5, 10, 15 and 30 days of storage at 30 ± 1°C and the AFB_1_ content of the samples was determined by UPLC/FLD.

### Inhibitory effect of AFB_1_ on growth of *P*. *eryngii*

The inhibitory effect of AFB_1_ on growth of *P*. *eryngii* was studied in agar media supplemented with a range of concentrations of AFB_1_, *i*. *e*. 60, 120, 250, 500 and 1000 ng/mL. The media used were MEA, MEA supplemented with 5% (w/v) wheat straw (MEAS), and MEA supplemented with 5% wheat straw and 2.5% (w/v) maize flour (MEASM).

Aliquots of a 1 mg/mL solution of AFB_1_ in toluene: acetonitrile (9:1, v/v) were added to melted MEA (50°C) to obtain the desired test dilutions (60 to 1000 ng/mL). The dilutions were thoroughly mixed and poured into 9 cm-diameter Petri dishes (12 ml per dish). For preparation of MEAS and MEASM, wheat straw obtained from a local dealer was fragmented in pieces ≤ 5 mm long and maize kernels were finely ground in a laboratory mill (mulino Cyclone, International PBI, Milano, Italy) to particles ≤ 0.2 mm; 0.5 g of straw and/or 0.25 g of ground maize were transferred into 2.5 cm-diameter and 15 cm long test tubes and autoclaved at 121°C for 30 min. After cooling, the glass tubes were filled with 12 ml of melted sterile MEA supplemented with the test dilutions of AFB_1_, thoroughly mixed and poured into 9 cm-diameter Petri dishes.

Then, each plate was inoculated with a 8 mm-diameter mycelial plug from a 20-day-old culture of *P*. *eryngii* on MEA and incubated at 30 ± 1°C in the dark for 30 days. Five replicates per each tested isolate were prepared. Control plates were prepared with *P*. *eryngii* on MEA, MEAS and MEASM not supplemented with AFB_1_. The growth of *P*. *eryngii* was assessed by the colony diameter of three representative isolates (ITEM 13681, ITEM 13697, ITEM 13688), measured with a ruler under a dissecting microscope every 48 hours.

### Degradation of AFB_1_ by *P*. *eryngii*

The capability of different isolates of *P*. *eryngii* to degrade AFB_1_ was tested in both liquid and solid cultures.

Liquid cultures of *P*. *eryngii* were prepared in MEB supplemented with the toxin. The assays were carried out in 12-well plates. Each well was filled with 2 mL of MEB containing 500 ng/mL of AFB_1_ and inoculated with a 8 mm-diameter mycelial plug from a 20-day-old culture of *P*.*eryngii* on MEA. Triplicate wells were prepared for each treatment and each sampling time and the cultures were incubated at 30 ± 1°C for 30 days in the dark. After 10, 20 and 30 days of incubation, 1 mL of culture medium was withdrawn from each well, filtered through 0.45-μm-pore-size cellulose filters (Labochem Science, Sant’Agata Li Battiati, Italy) and stored at -20°C until the analysis. Five hundred microliters of each sample were diluted with 500 μl of ultrapure water produced by a Milli-Qsystem (Millipore, Bedford, MA, USA), filtered through 0.2-μm-pore-size regenerated cellulose (RC) filters (Grace, Deerfield, IL, USA) and 10 μL of the filtrate were injected directly into the UPLC apparatus through a full loop injection system. The percent degradation (D) was calculated by the formula D (%) = [(C_i_−C_f_)/ C_i_] x 100, where C_i_ was the concentration of AFB_1_ in the non-inoculated control and C_f_ was the concentration of AFB_1_ in culture filtrates 10, 20 and 30 days post inoculation of *P*. *eryngii*.

Solid cultures of *P*. *eryngii* were grown on MEASM supplemented with 500 ng/mL of AFB_1_. The medium was prepared and inoculated as described above. Non-inoculated plates containing the medium supplemented with AFB_1_ were used as controls. After 15 and 30 days of growth, six 10 mm-diameter plugs of culture were excised with a cork-borer along one radius of the colony, at regular distances from the initial inoculation point to the edge of the colony, and transferred to a test tube. The samples (apx. 1 g) were precisely weighted and extracted with 5 mL of a methanol-water (80:20, v/v) solution in a KS 4000i orbital shaker (IKA Werke GmbH & Co. KG., Staufen, Germany) at 250 rpm for 60 min. at room temperature. Five hundred microliters of each extract were then diluted, processed and analyzed for AFB_1_ by UPLC/FLD as described for liquid cultures. The percent degradation (D) was calculated like for liquid cultures.

### Cultivation of *P*. *eryngii* in a AFB_1_-contaminated mushroom medium

*Pleurotus eryngii* was cultivated on a substrate similar to that used for production of commercial mushrooms, which contained ground maize spiked with aflatoxin. To produce AFB_1_, the aflatoxigenic *A*. *flavus* strain ITEM 7764 was cultured on potato-dextrose-agar (PDA) for 7 days at 25°C in the dark; a conidial suspension in sterile distilled water was prepared from the PDA cultures and used to inoculate 200 g of autoclaved (121°C for 30 min.) maize kernels in 1000 mL Erlenmeyer flask, to reach a final concentration of 1 × 10^4^ conidia/g. Flasks were incubated for 21 days at 25°C in the dark and analyzed for aflatoxin content. To extract aflatoxins, 300 mL methanol-water (80:20, v/v) were added into each flask, shaken on a rotary shaker for 60 min. at 250 rpm and filtered through Whatman No. 4 filter paper. Five hundred microliters of extract were diluted with 500 μL of water, filtered through RC 0.20 μm filters and 10 μL of the extract was injected into the UPLC apparatus for aflatoxin quantification. The extract was used to spike non contaminated ground maize to have the final concentration of 500 μg/kg of maize.

The substrate used for cultivation of *P*. *eryngii* contained 50% wheat straw, 25% spiked maize, 12.5% sugar beet and 12.5% field beans (*Vicia faba minor*). All the raw materials were obtained from a local supplier (Gruppo I.F.E srl, Matera, Italy). Wheat straw was fragmented in pieces about 2–3 mm long, sugar beet and field beans were ground in a blender Sorvall Omnimixer (Dupont Intruments, Newton, CT, USA) for 2 min. Magenta^™^ vessels (Sigma, 77 × 77 ×77 mm) were filled with 18 g of growth substrate with 1% (w/w) of calcium carbonate. Dry ingredients were carefully mixed and 32 mL of tap water were added to reach approx. 65% (w/w) of moisture content; the mix was finally autoclaved at 121°C for 60 min. Once cooled, the substrate was inoculated with 3 g of spawn of *P*. *eryngii* ITEM 13681, prepared as described by Estrada and co-workers [27], with some minor modifications. Briefly 100 g of durum wheat kernels were mixed with 70 mL of warm tap water in Magenta vessels and then incubated for 28 days at 30 ± 1°C in the dark. The AFB_1_ content in the growth substrate was assessed by UPLC/FLD 0, 7, 14, 21, and 28 days post inoculation (d.p.i.), in triplicate vessels. Non-inoculated vessels containing aflatoxin-contaminated substrate were used as controls.

In order to assess the possible carry-over of AFB_1_ or its metabolite AFOL in the basidiocarps, 28 d.p.i. vessels were opened, covered with a thin layer (1–2 cm) of soil and placed in greenhouse at 26°C/ 21°C day/night with a 12 h photoperiod to promote the fruiting of carpophores. The cultures were maintained constantly moist by nebulization of moderate amounts of water. Mushrooms were harvested after 15 additional days of growth, when the basidiomata was ripe and the mushroom cap was flat. Fresh mushrooms from each vessel were individually weighed to determine the yield and the biological efficiency (BE) as the ratio fresh mushroom weight/dry weight of the substrate, expressed as percentage. The spent substrate and the basidiocarps were analyzed for AFB_1_ and AFOL. Cultures prepared with non-contaminated maize were used as controls. The experiment was performed in triplicate and repeated once.

### Determination of AFB_1_ and AFOL in spent mushrooms substrate and basidiocarps of *P*. *eryngii*

The aflatoxin-contaminated growth substrate of *P*. *eryngii* of each vessel was dried at 50°C until constant weight. A portion of 20 g of substrate from each replicate was extracted with 100 mL of acetonitrile-water (84:16, v/v) by blending at high speed for 3 min with a Sorvall Omnimixer. The extract was filtered through Whatman No. 4 paper filters and 50 μL of acetic acid were added. Aliquots of 8 mL were purified with Mycosep^®^ 224 AflaZon column (Romer Labs). The extract was diluted 1:1 with pure water and 10 μL were injected into the UPLC apparatus [28]. The percent degradation (D) of AFB_1_ in *P*. *eryngii* growth substrate was calculated like for liquid cultures.

To investigate the carry-over of AFB_1_ and AFOL in the basidiocarps, 2 g of dry fruit bodies from each replicated vessel were extract in a Sorvall Omnimixer for 3 min with 50 mL of acetonitrile-water (84:16, v/v). The extract was then filtered, processed and analyzed as described for the growth substrate above.

### Chemicals

AFB_1_ and AFOL chemical standards (purity > 99%) were supplied by Sigma-Aldrich (Milan, Italy). All solvents (HPLC grade) were purchased from VWR International Srl (Milan, Italy). Water Millipore Milli-Q system (Millipore, Bedford, MA, USA). Mycosep^®^ 224 AflaZon column were obtained from (Romer Labs^®^, Getzersdorf, Austria). Paper filters Whatman no. 4 was obtained from Whatman (Maidstone, UK) and RC 0.2 μm (regenerated cellulose membranes) filter were obtained from Grace.

### Statistical analysis

Data were analyzed by one-way analysis of variance (ANOVA) and Tukey–Kramer multiple comparison test. The statistical analyses were performed using the GraphPad Instat 3.0 software (GraphPad Software, San Diego, CA).

## Results

### Chemical stability of AFB_1_

Experiments to test stability of AFB_1_ in MEB showed no significant degradation in respect to control after 5, 10, 15 and 30 days at 30 ± 1°C in the dark. The experiment was highly reproducible (coefficient of variation = 97.2 ± 3.5%).

### Inhibitory effect of AFB_1_ on growth of *P*. *eryngii* in agar medium

**Fig 1:**
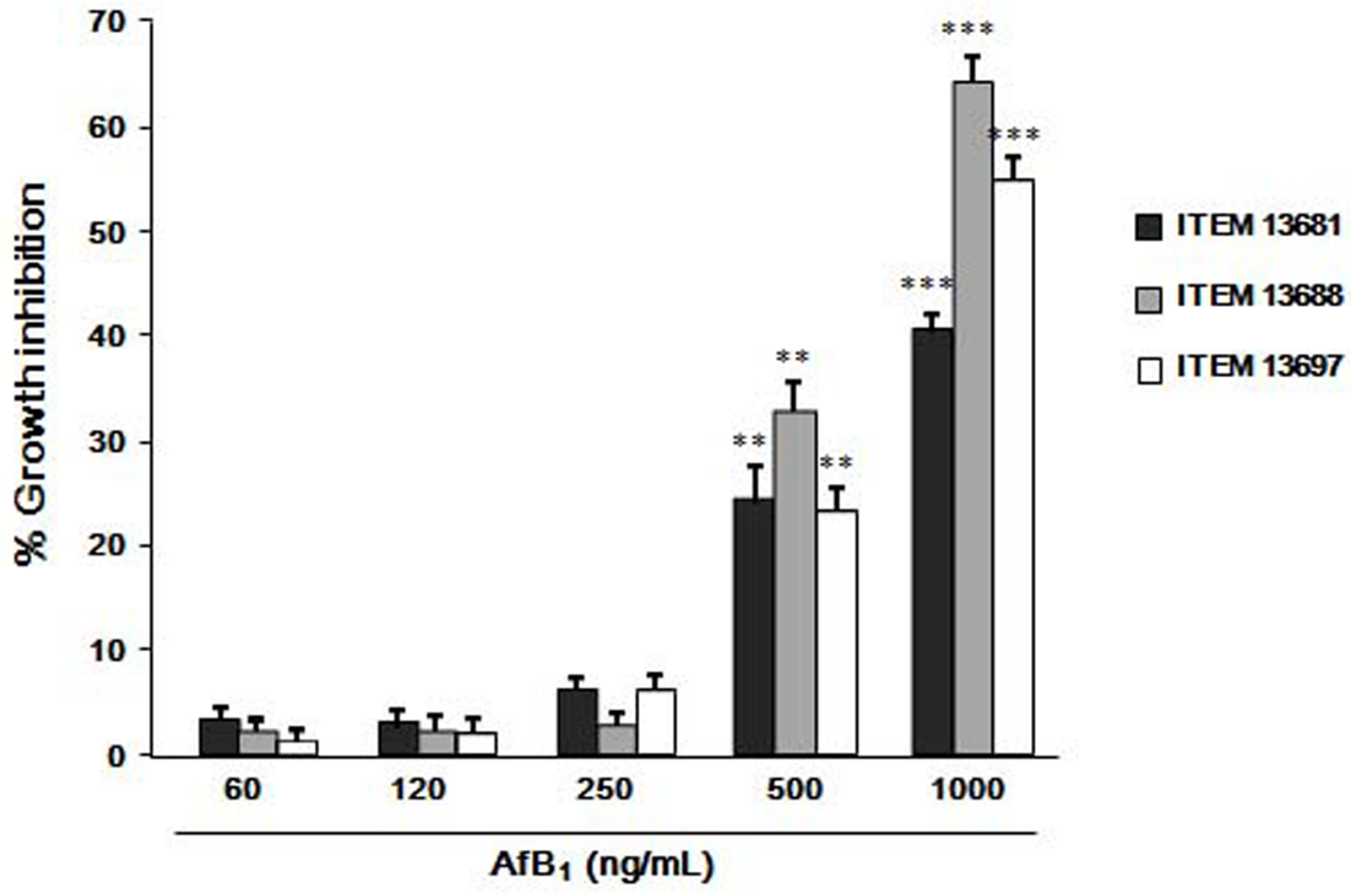
Inhibitory effect of AFB_1_ on growth of *P*. *eryngii*. The isolates ITEM 13681, ITEM 13688 and ITEM 13697 were grown for 15 days at 30 ± 1°C in the dark on malt extract agar (MEA) containing different concentrations (60, 120, 250, 500, 1000 ng/mL) of AFB_1_. Data are the means ± SD (n = 5) of the percent reduction in colony diameters with respect to control. Statistically significant differences with control are indicated by asterisks (*** = *P* < 0.001, ** = *P*<0.01; One-way Anova).

Although the three isolates showed a different sensitivity to AFB_1_, all exhibited a statistically significant growth inhibition on MEA when exposed to 500 ng/mL or higher concentration of AFB_1_ (Fig 1). Concentrations of AFB_1_ of 250 ng/mL or lower did not significantly affect the mycelial growth; *P*. *eryngii* ITEM 13688 was the most sensitive isolate, showing 60 ± 2% and 30 ± 2% of growth inhibition when exposed for 15 days to 1000 and 500 ng/mL of AFB_1_, respectively; *P*. *eryngii* ITEM 13681 was the most tolerant isolate, showing a growth inhibition of 37± 1% and 20 ± 3% at 1000 and 500 μg/mL of AFB_1_ respectively (Fig 1).

**Fig 2:**
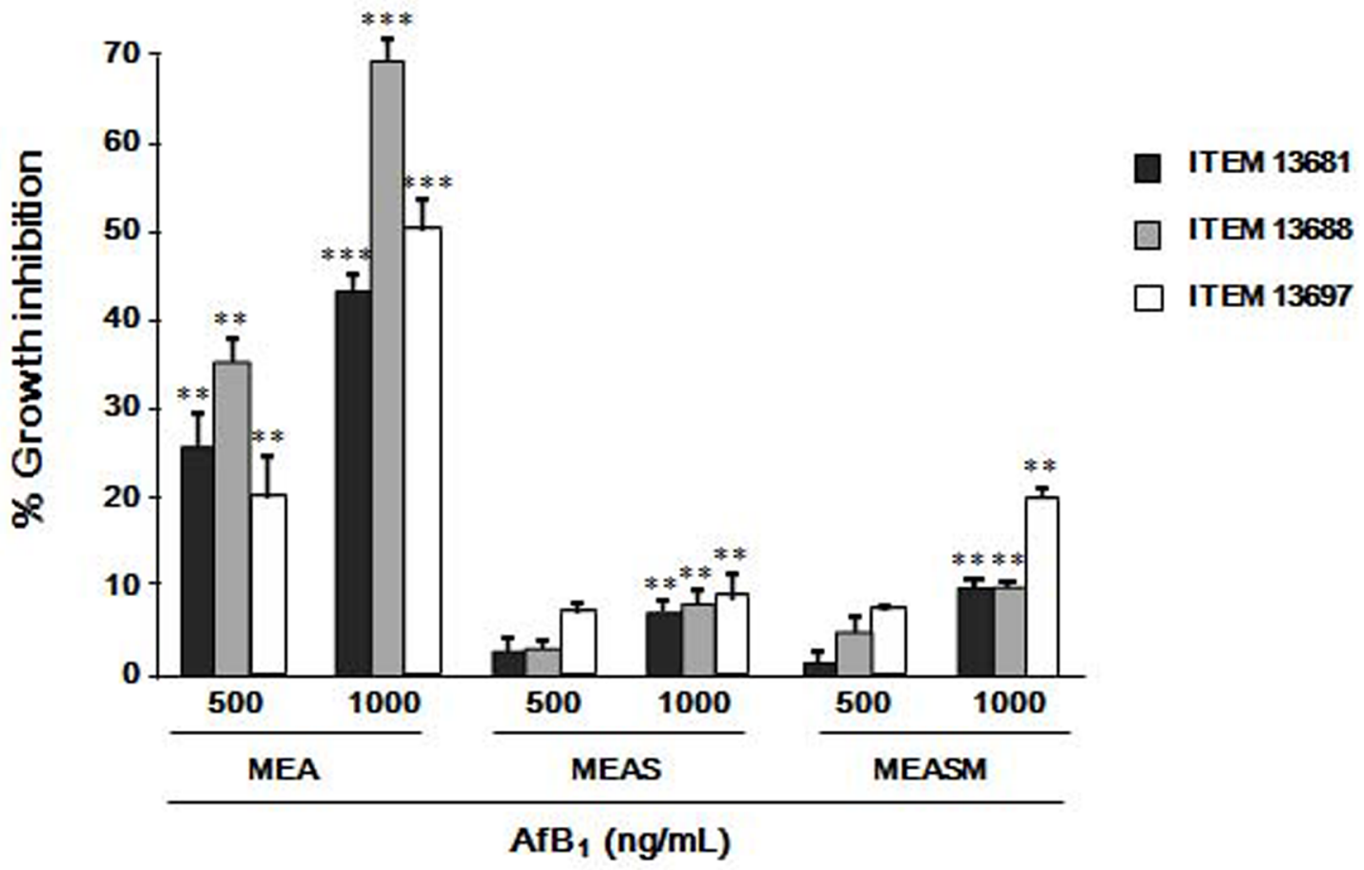
Reduction of the inhibitory effect of AFB_1_ on *P*. *eryngii* in the presence of wheat straw. The isolates ITEM 13681, ITEM 13688 and ITEM 13697 were grown on malt extract agar (MEA), MEA supplemented with 5% (w/v) wheat straw (MEAS) and MEA supplemented with 5% wheat straw and 2.5% (w/v) maize flour (MEASM) containing 500 and 1000 ng/mL of AFB_1_. Data are the means ± SD (n = 5) of the percent reduction in colony diameters with respect to controls after 15 days of growth at at 30 ± 1°C in the dark. Statistically significant differences with control are indicated by asterisks (*** = *P* < 0.001, ** = *P*<0.01; One-way Anova).

For all the isolates tested, the inhibition of growth on MEAS and MEASM was lower than on MEA in the presence of 1000 ng/mL of AFB_1_ and not statistically significant at the concentrations of 500 ng/mL or lower (Fig 2).

### Degradation of AFB_1_ by *P*. *eryngii*

Degradation of AFB_1_ in the liquid medium (MEB) are shown in Table 1. After 10 days of growth the isolates ITEM 13730, ITEM 17015 and ITEM 13662 degraded 99% of AFB_1_. The isolates ITEM 13682 and ITEM 13696 degraded AFB_1_ by 95% and 94%, respectively, and the isolates ITEM 13681, ITEM 13697 and ITEM 13676 showed approximately 90% degradation. The isolate that showed the lowest degradative capability (80%) was ITEM 13688. However, after 20 days of incubation the differences among the strains were not statistically significant and after 30 days all the isolates totally degraded AFB_1_.

**Table 1:** Degradation of AFB_1_ by strains of *P*.*eryngii* grown in malt extract broth (MEB) supplemented with 500 ng/mL of AFB_1_, after 10, 20 and 30 days of incubation at 30 ± 1°C in the dark.

Isolate	10 Days			20 Days			30 Days		
	AFB_1_^(^^a^^)^ (ng/mL)		D^(^^b^^)^ (%)	AFB_1_^(^^a^^)^ (ng/mL)		D^(^^b^^)^ (%)	AFB_1_^(^^a^^)^ (ng/mL)		D^(^^b^^)^ (%)
**Control**	518 ± 18	A		501 ± 22	A		519 ± 22	A	
**ITEM 13662**	6 ± 1	F	99	1 ± 0.1	B	100	1 ± 0.1	B	100
**ITEM 13676**	47 ± 5	CD	91	4 ± 0.3	B	99	1 ± 0.2	B	100
**ITEM 13681**	47 ± 2	CD	91	17 ± 2	B	97	3 ± 0.4	B	100
**ITEM 13682**	28 ± 7	DF	95	2 ± 0.5	B	97	0 ± 0.2	B	100
**ITEM 13688**	98 ± 7	B	81	22 ± 6	B	96	3 ± 0.7	B	100
**ITEM 13696**	30 ± 5	DE	94	9 ± 0.3	B	98	1 ± 0.2	B	100
**ITEM 13697**	59 ± 11	C	89	5 ± 1	B	99	1 ± 0.1	B	100
**ITEM 13730**	11 ± 2	EF	98	3 ± 0.2	B	99	1 ± 0.1	B	100
**ITEM 17015**	6 ± 0.5	F	99	1 ± 0.1	B	100	1 ± 0.2	B	100

^a^ Concentration of AFB_1_ detected in the culture filtrate. Data represent the mean values of AFB_1_ in 3 replicated cultures ± SD. Values in column followed by different letters are significantly different for *P* < 0.001 (Tukey–Kramer Multiple Comparison Test).

^b^ Percent degradation of AFB_1_ calculated as: D (%) = [(C_i_−C_f_)/ C_i_] x 100, where C_i_ was the concentration of AFB_1_ in the non-inoculated control and C_f_ was the concentration of AFB_1_ in filtrates of *P*. *eryngii* cultures at the given time.

Degradation of AFB_1_ by the *P*. *eryngii* isolates grown on the agar medium (MEASM) was analyzed in 15 and 30 days cultures (Table 2). After 15 days of incubation, all the strains significantly degraded the AFB_1_ supplemented to the medium, in percentages that ranged from 43 to 59%. After 30 days the degradation of AFB_1_ by the strain ITEM 17015 increased to 84%, and that of the strains ITEM 13696 and ITEM 13662 were 83 and 82% respectively. The strains ITEM 13688 and ITEM 13676, the least effective strains in degradation of AFB_1_, degraded AFB_1_ in the medium by 71 and 65% respectively.

**Table 2:** Degradation of AFB_1_ by *P*.*eryngii* grown on malt extract-agar plus wheat straw and maize flour (MEASM) supplemented with 500 ng/mL of AFB_1_, after 15 and 30 days of incubation at 30 ± 1°C in dark.

Isolate	15 Days			30 Days		
	AFB_1_^(^^a^^)^ (ng/mL)		D^(^^b^^)^ (%)	AFB_1_^(^^a^^)^ (ng/mL)		D^(^^b^^)^ (%)
**Control**	419 ± 5	A		436 ± 6	A	
**ITEM 13662**	183 ± 5	BC	56	74 ± 2	D	82
**ITEM 13676**	192 ± 5	BC	54	147 ± 7	B	65
**ITEM 13681**	215 ± 20	BC	49	88 ± 6	C	79
**ITEM 13682**	204 ± 8	BC	51	86 ± 1	C	79
**ITEM 13688**	237 ± 5	B	43	120 ± 13	B	71
**ITEM 13696**	185 ± 13	BC	55	69 ± 12	D	83
**ITEM 13697**	192 ± 26	BC	54	119 ± 6	BC	72
**ITEM 13730**	188 ± 13	BC	59	79 ± 11	CD	81
**ITEM 17015**	173 ± 9	C	59	66 ± 7	D	84

^a^ Concentration of AFB_1_ detected in the culture medium. Data represent the mean values of AFB_1_ in 3 replicated cultures ± SD. Values in column followed by different letters are significantly different for *P* < 0.001 (Tukey–Kramer Multiple Comparison Test).

^b^ Percent degradation of AFB_1_ calculated as: D (%) = [(C_i_−C_f_)/ C_i_] x 100, where C_i_ was the concentration of AFB_1_ in the non-inoculated control and C_f_ was the concentration of AFB_1_ in *P*. *eryngii* cultures at the given time.

### Mushroom cultivation on aflatoxin-contaminated substrate

**Fig 3:**
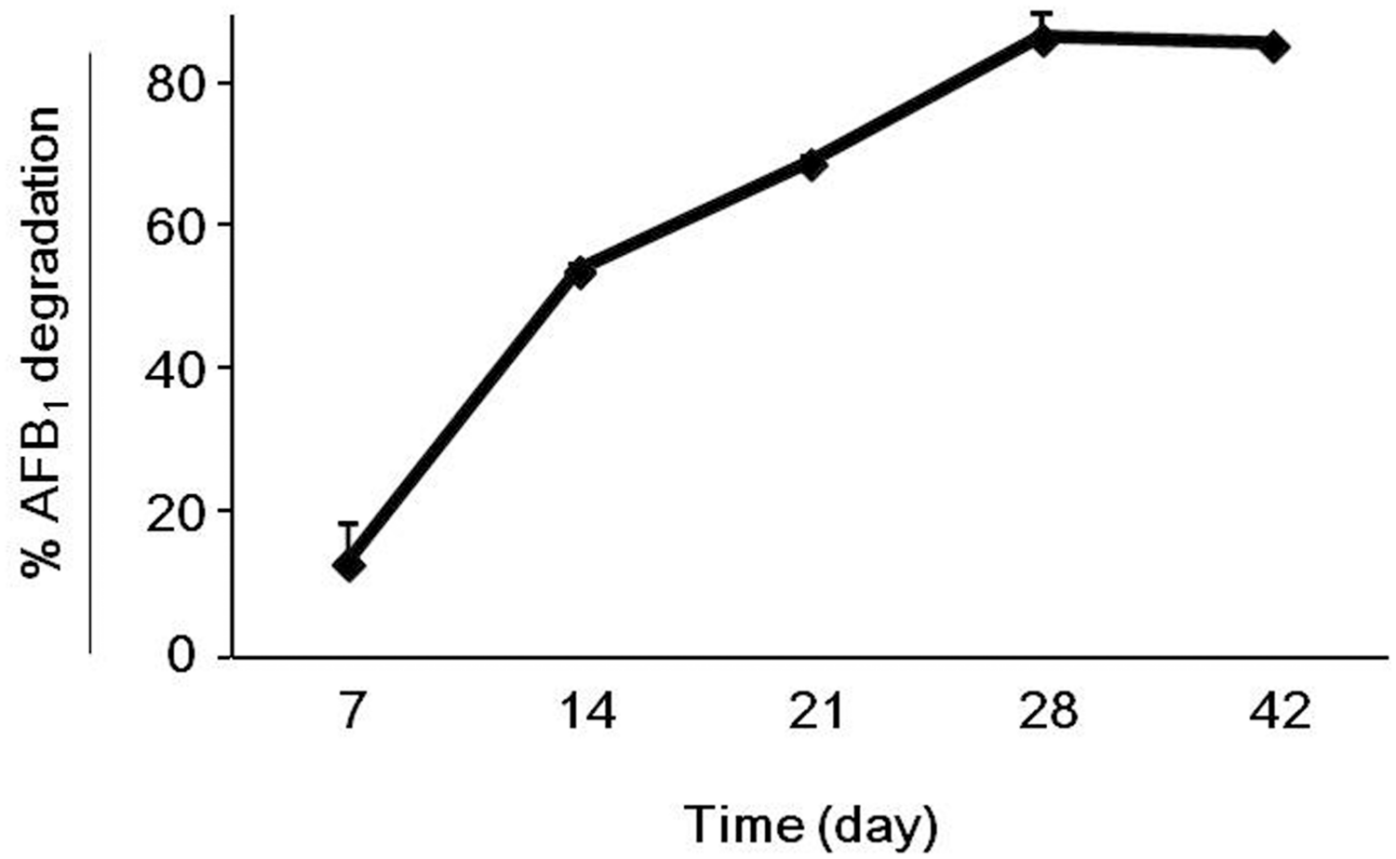
Time course of degradation of AFB_1_ by *P*.*eryngii* ITEM 13681. The isolate ITEM 13681 was cultivated on a mushroom growth substrate that contained 128 ± 6 μg/kg of AFB_1_. Data are the means ± SD (n = 3) of the percent AFB_1_ degradation with respect to the control.

**Fig 4:**
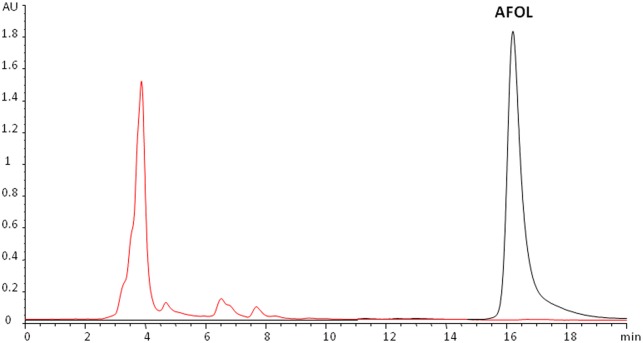
Determination of AFOL in the biomass of *P*. *eryngii*. Overlay of HPLC/FLD chromatograms of a standard solution of aflatoxicol (AFOL, black line) and the extract of *P*. *eryngii* biomass developed on the contaminated mushroom substrate (red line). AFOL was not present in the extract.

The artificially contaminated cultivation substrate contained 128 ± 6 μg/kg of AFB_1_ at the time of inoculation with *P*. *eryngii* ITEM 13681 (day 0). The percentages of degradation of AFB_1_ at 7, 14, 21, and 28 d.p.i. are shown in Fig 3. Seven d.p.i. degradation was 13 ± 6% and 28 d.p.i. the level of degradation reached the maximum value of 86 ± 0.2% that did not change significantly until the end of the experiment (42 d.p.i). AFOL was not detected in the fungal biomass developed in the contaminated mushroom substrate (Fig 4). The analysis of basidiocarps of *P*. *eryngii* obtained on the contaminated mushrooms substrate did not reveal the presence of either AFB_1_ or AFOL.

**Fig 5:**
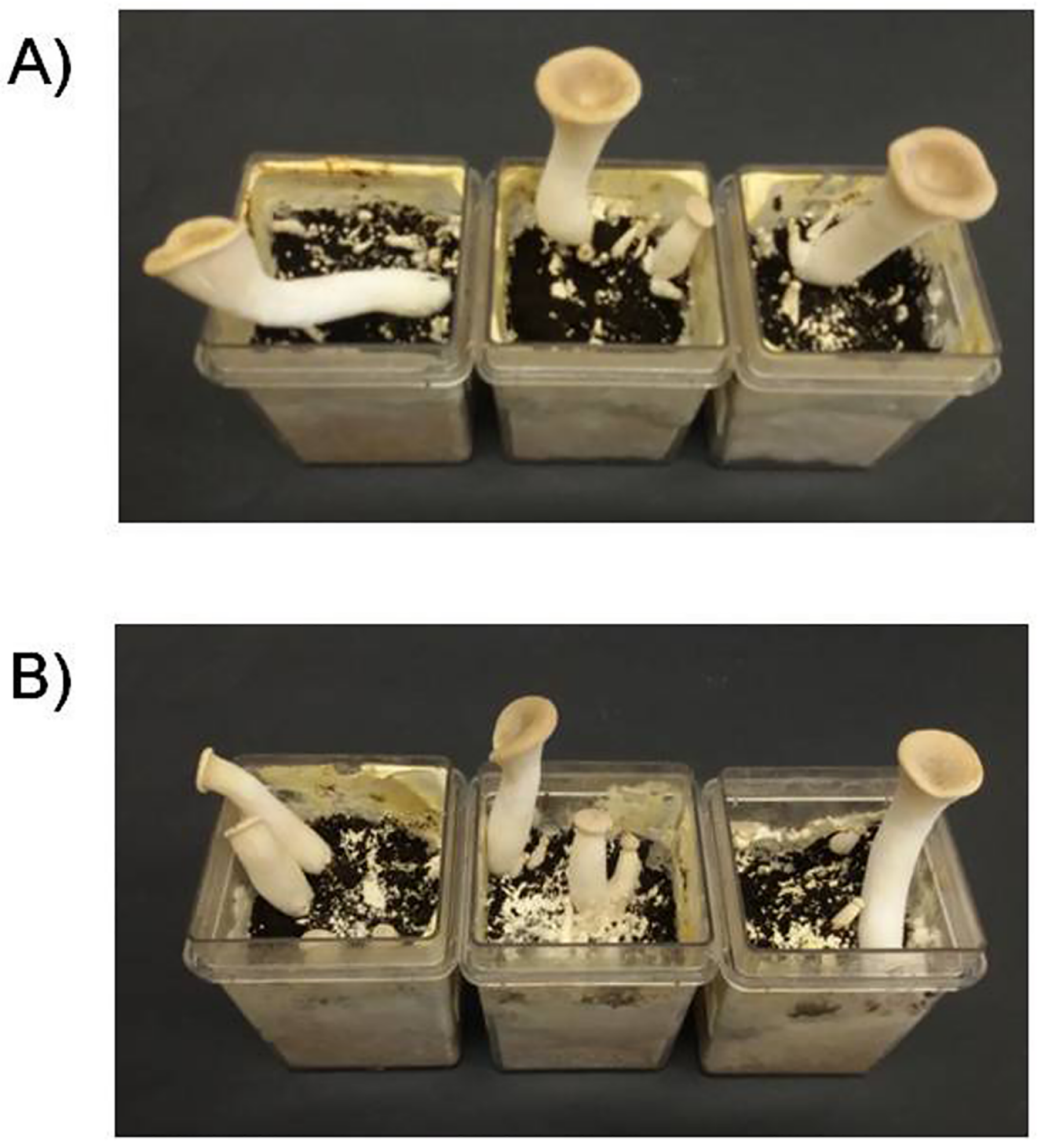
*Pleurotus eryngii* cultivated in a AFB_1_-contaminated mushroom medium. Production of basidiocarps (fruit bodies) by *P*.*eryngi*i ITEM 13681 in a mushroom cultivation medium; A) medium contaminated with 128 μg/kg of AFB_1_; B) non-contaminated control. In both the conditions *P*. *eryngii* ITEM 13681 produced well-developed basidiocarps, as well as growing immature fruit-bodies and fruit primordials in 42 days.

In order to evaluate a possible effect of AFB_1_ on production of basidiocarps, yield and BE were assessed when the basidiocarps were fully ripe and the mushroom cap was flat (Fig 5). Yield and BE of *P*. *eryngii* ITEM 13681 grown on the AFB_1_ contaminated substrate were 9 ± 1 grams and 72 ± 11% respectively, not significantly different from values of the control cultures (9 ± 0,8 g; 70 ± 5%).

## Discussion

Previous research has proven the capability of some white-rot fungi to detoxify aflatoxins. In particular the genus *Pleurotus*, which comprises edible cultivable mushrooms such as *P*. *ostreatus* and *P*. *eryngii*, produce the enzymes laccase and peroxidase that have been proved to degrade aflatoxins [20–23, 29, 30].The aim of this work was to investigate the AFB_1_-degrading capability of the species *P*. *eryngii* and explore the potential of this mushroom for development of practical technologies aimed at recovery and valorization of aflatoxin-contaminated cereal wastes.

Aflatoxin B_1_ has a dose dependent inhibitory effect on the growth of *P*. *eryngii*. The tolerated level of AFB_1_ varies among *P*. *eryngii* isolates and depends on the substrate composition. It is not clear if the individual variation in the tolerance to AFB_1_ is due to differences in mycelium sensitivity or to different levels in AFB_1_-degrading capability of the strains. The latter hypothesis seems more likely, as the most affected isolate (ITEM 13688, Fig 1) was also the least effective in AFB_1_ degradation (Tables 1 and 2). The presence of 5% wheat straw in the growing medium doubled (from 250 to 500 ng/mL) the concentration of AFB_1_ that was tolerated without resulting in any significant growth inhibition (Figs 1 and 2). The ligninolytic enzymes involved in AFB_1_ detoxification are inducible enzymes [31–33], therefore the higher tolerance to AFB_1_ shown by *P*. *eryngii* when grown of wheat straw may be conceivably due to the increase in the synthesis of such enzymes that is induced by the lignocellulosic materials in the culture medium.

Differences in the AFB_1_-degrading capability among the nine tested *P*. *eryngii* strains were found both in liquid culture on a semi-synthetic medium, (MEB) and on an agar medium supplemented with wheat straw and maize (MEASM) (Tables 1 and 2), even if the differences among the isolates were not dramatic. In liquid medium, where the degrading enzymes can diffuse freely, the levels of degradation were quite high (81 ± 1.4 to 99 ± 0.2%) already after 10 days of growth, to reach 100% in all the tested strains after 30 of growth. On the contrary, in the agar medium, where the enzymes presumably diffused more slowly through the agar layer, AFB_1_ was degraded by 71–94% after 30 days of growth. Based on these results, the capability to degrade AFB_1_ exhibited by *P*. *eryngii* appears to be widely distributed in members of the species. Individual differences in the efficiency of degradation, however, exist and might be due to differences in the kinetics of biosynthesis of single enzymes, in the composition of the enzyme pool implicated in AFB_1_ degradation (laccase and Mn-peroxidases), or in the degradative efficiency of different enzyme isoforms [29].

One more goal of our work was the exploitation of the AFB_1_-degrading capability of *P*. *eryngii* for the development of a technology aimed at the recovery and valorization of contaminated cereals. So far, cultivation of mushrooms is the only biotechnological process that allows for bioconversion of lignocellulosic organic waste into protein-rich and high nutritional value nutriment, thus contributing to reduction of environmental pollution [34]. We have demonstrated that this technology may also be applied to the recycling of aflatoxin-contaminated cereals, which should be otherwise destroyed or directed to alternative uses, such as production of bioethanol. In a laboratory-scale cultivation on a growth medium that is routinely used in mushroom farms, the isolate *P*. *eryngii* ITEM 13681 was able to bioconvert up to 86% of the AFB_1_ in the medium (128 μg/kg) in 28 days. Although the mushroom growth medium contained 25% (w/w) of maize contaminated with 500 ppb of AFB_1_, *P*. *eryngii* did not show any significant reduction of either biological efficiency or mushroom yield.

AFOL is a derivative of AFB_1_ that originates from reduction of the cyclopentanone carbonyl of the coumarine moiety [35]. The biological conversion of AFB_1_ to AFOL by intracellular enzymes has been demonstrated for several fungi [35, 36]. Although AFOL is 18 times less toxic than AFB_1_ [35], it maintains a significant toxicity and can be reconverted to AFB_1_ [36], thus still representing a safety risk. In this study AFOL was not found, as a by-product of enzymatic or pH-dependent reduction of AFB_1_ by *P*. *eryngii*, in the fungal biomass. Also, the mature basidiocarps did not contain detectable levels of either AFB_1_ or AFOL, thus ruling out translocation or "carry-over" of these toxins through the fungal thallus.

In conclusion, our results show that *P*. *eryngii* is able to degrade aflatoxin B_1_ in both liquid and solid media. The biodegradation of AFB_1_-contaminated cereals by *P*. *eryngii* that we have demonstrated to occur in a laboratory-scale mushroom cultivation needs appropriate validation prior to become a practical technology. Nevertheless our results highlight that it may be regarded as a candidate process for bioconversion of contaminated staple cereals into valuable products intended for animal feeding. These findings make a contribution towards the development of preventative strategies to reduce AFB_1_ contamination of feeds and animal-derived foods. Further research will be aimed at the identification of degradation products of aflatoxins and to the assessment of the technical and economic sustainability of remediation of aflatoxin-contaminated commodities through mushroom bioconversion into non-toxic feeds.
